# Associations between COVID-19 related media consumption and symptoms of anxiety, depression and COVID-19 related fear in the general population in Germany

**DOI:** 10.1007/s00406-020-01171-6

**Published:** 2020-07-20

**Authors:** Antonia Bendau, Moritz Bruno Petzold, Lena Pyrkosch, Lea Mascarell Maricic, Felix Betzler, Janina Rogoll, Julia Große, Andreas Ströhle, Jens Plag

**Affiliations:** Department of Psychiatry and Psychotherapy, Campus Charité Mitte, Charité - Universitätsmedizin Berlin, corporate member of Freie Universität Berlin, Humboldt-Universität zu Berlin, and Berlin Institute of Health, Charitéplatz 1, 10117 Berlin, Germany

**Keywords:** SARS-CoV-2, Corona, Pandemic, Mental health, Psychological distress, Media coverage

## Abstract

In context of the current COVID-19 pandemic the consumption of pandemic-related media coverage may be an important factor that is associated with anxiety and psychological distress. Aim of the study was to examine those associations in the general population in Germany. 6233 participants took part in an online-survey (March 27th–April 6th, 2020), which included demographic information and media exploitation in terms of duration, frequency and types of media. Symptoms of depression, unspecific anxiety and COVID-19 related anxiety were ascertained with standardized questionnaires. Frequency, duration and diversity of media exposure were positively associated with more symptoms of depression and unspecific and COVID-19 specific anxiety. We obtained the critical threshold of seven times per day and 2.5 h of media exposure to mark the difference between mild and moderate symptoms of (un)specific anxiety and depression. Particularly the usage of social media was associated with more pronounced psychological strain. Participants with pre-existing fears seem to be particularly vulnerable for mental distress related to more immoderate media consumption. Our findings provide some evidence for problematical associations of COVID-19 related media exposure with psychological strain and could serve as an orientation for recommendations—especially with regard to the thresholds of critical media usage.

## Introduction

During the last months, the COVID-19 pandemic has grown to the most serious international health problem of the last decades and therefore represents a substantial challenge for communities all over the world. In addition to the immediate threat posed by the virus, the current situation also comes along with significant distress as well as substantial fears and worries related to its social and economic consequences for a significant number of people [1–3]. The psychological strain on the general population, however, may be further increased by the requirements of quarantine or the so-called “social distancing” that is supposed not only to contain the spreading of the infection but also to profoundly impede the interpersonal communication.

In this context, the role of the media has become quite essential on different levels. First, they are important sources of information with regard to topics (in)directly related to the virus (e.g., infection rates, measures of the government, recommendations of the public health authorities, or the economic and social situation). Several studies demonstrated the meaning of media coverage during previous pandemics since the type, as well as the frequency of news reporting, was shown to significantly influence individuals’ health-related attitudes and behavior [4–6]. In addition, particularly social media may further hold the potential to (partly) bridge the problems resulting from the restrictions of the face-to-face contact. In these times platforms like Facebook, Twitter, Instagram or specific internet forums are essential ways to share opinions, personal experiences, moments of happiness, worries or fears for many people as reflected by a dramatically increase of COVID-19-related terms up to several million mentions on these channels until March 2020 [7]. This way of communication therefore may have an important function for psychological wellbeing and applying social media for staying in contact with significant persons is decidedly recommended by official sites in Germany [8].

But these positive aspects might be just one side of the medal. The exploitation of media may also bear a certain risk for the mental health state due to elevated stress levels, particularly once a critical mass of predominantly negative news or unfiltered information is reached. Being aware of this, the World Health Organization (WHO) currently created the term “infodemic” in the context of the present crisis for an “over-abundance of information—some accurate and some not—that makes it hard for people to find trustworthy sources and reliable guidance when they need it” [9]. To “help minimize fears” the WHO, but also other international operating organizations therefore published distinct mental health considerations. Common central points of these recommendations encompass the restriction of (distress-inducing) media usage with respect to its duration and frequency as well as being kept informed using official websites of (inter)national health authorities to “distinguish facts from rumours” [10–12]. These reservations regarding media consumption related to a macrosocial disaster are backed up by existing data in this field. Former trials reported about significant psychological strain or distinct mental conditions resulting from crisis-related media coverage [13–15] and individual vulnerability was suspected to account significantly for this risk [16]. In the context of the current pandemic, up-to-date data also suggest that topic-specific media reporting results in a high level of stress in the vast majority of people [17]. However, we are aware of only one study that addressed the influence of COVID-19 related media exposure on stress-related mental symptoms so far. Including more than 4000 adult citizens, this web-based approach found the frequency of social media exposure to be significantly positively correlated to symptoms of depression and anxiety within the general population in China [18].

To further elucidate the relationship between media consumption and symptoms of depression, unspecific anxiety, as well as COVID-19 specific fear in western communities, we also performed a web-based investigation within the general population in Germany. In our analyses, we firmly considered not only the impact of the frequency and duration of media exploitation per day but also the meaning of specific types of media on these stress-related domains. Since it should be quite important at a practical level to define risky media usage as precise as possible, we further thought to determine concrete numeric values with respect to the daily frequency/duration of distress-inducing media exposure. Finally, we investigated whether there is a specific risk of psychological distress related to media consumption in individuals who already suffered from health-related fears to gain an insight into susceptible vulnerability factors.

## Methods

### Design

The present study has a cross-sectional, observational design, using the first wave of data of a longitudinal online-survey in a convenience sample of the general population in Germany. Prior to recruitment, the study was approved by the ethics committee of Charité Universitätsmedizin Berlin (EA1/071/20) and registered on clinicaltrials.gov (NCT04331106).

### Recruitment

**Fig. 1:**
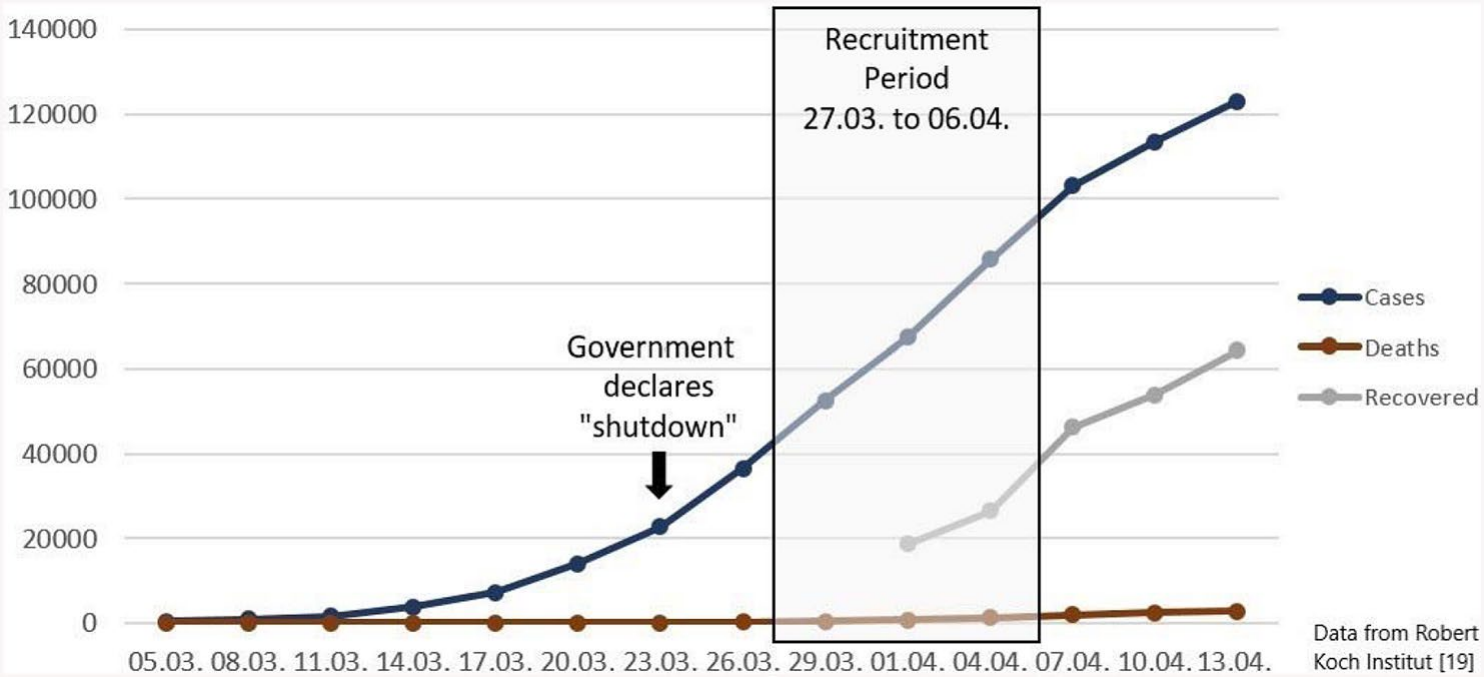
The situation in Germany regarding COVID-19 during recruitment in terms of infected cases, number of deaths and number of recovered persons

An online self-report questionnaire via *SoSci Survey* was used to survey the impact of media consumption regarding the COVID-19 pandemic on anxiety and depressive symptoms. The data collection of the first wave took part from March 27th, 2020 (when in Germany 42,288 cases of infection and 253 deaths attributed to COVID-19 were reported; see Fig. 1) to April 6th, 2020 (95,391 infections and 1434 deaths) [19]. Shortly before the recruitment period started, strict restrictions became effective nationwide to reduce the exponentially rising numbers of infection (e.g., physical distancing and closure of most institutions and shops).

Primarily social media platforms (Twitter, Facebook, Instagram), news portals, and the webpage of the Charité were used for recruitment. Prior to participation, all participants gave informed consent and approximately 10–15 min were needed to complete the entire online survey.

### Eligibility criteria

The inclusion criteria were the minimum age of 18 years, the current residence in Germany, and the ability to complete the questionnaire in German. Other inclusion or exclusion criteria did not apply.

### Assessment

The survey contained questions about demographic information (such as age, gender, and educational level) and personal experiences with the virus (e.g., being in quarantine, being tested or diagnosed for SARS-CoV-2, etc.). The daily average of hours of media usage to inform oneself about COVID-19 as well as the daily average of times of media exploitation was obtained. Furthermore, the questionnaire included two items regarding the self-reflection of the impact of media consumption: “I think I should reduce media consumption related to Corona because it is very stressful for me” and “I already reduced media consumption related to Corona because it was very stressful for me”. Both items were rated on a 6-point Likert scale, ranging from 1 (“not true at all”) to 6 (“totally true”).

To assess specific anxiety symptoms regarding the COVID-19 pandemic, a modified version of the validated DSM-5 Severity-Measure-For-Specific-Phobia-Adult-Scale [20, 21] was used. The scale includes ten items, rated on a 5-point Likert scale ranging from 0 (“never”) to 4 (“all the time”). The sum score of the ten items can be considered to classify the severity of the phobic symptoms in: none (0–4) mild (5–14), moderate (15–24), severe (25–34), and extreme (35–40).

The validated ultra-brief screening scale of the Patient Health Questionnaire-4 PHQ-4; [22, 23] was included to screen for general anxiety and depressive symptoms. The PHQ-4 consists of a general anxiety subscale (GAD-2) and a depression subscale (PHQ-2) of each two items. All four items add up to the PHQ-4 total score. A 4-point Likert scale ranging from 0 (“not at all”) to 3 (“nearly every day”) was used to rate the intensity of the items. The PHQ-4 sum score is classified in none (0–2), mild (3–5), moderate (6–8) and severe (9–12) symptoms of general/unspecific anxiety and depression.

### Data analysis

IBM SPSS Statistics Version 25 was used for all analyses and the significance-level was set to 0.05 (two-tailed). The online-questionnaire included eight pages. For the analyses, only participants that at least completed page seven were included (*N* = 6180). 93.6% of the sample (*N* = 5721) completed all eight pages. Average percentage of missing data on item-level was 1.2% (range 0.0–16.3%). Missing data were handled by casewise-deletion. Descriptive statistics, Pearson’s correlations and analyses of variance were used for data analysis. For the effects of age and gender has been controlled in all analyses.

On a continuum regarding the quality and reliability of information, social media can be assumed rather low; as a source of unfiltered information with a high risk for “fake-news” and “scaremongering” [9]. In contrast, official websites of the government and health authorities provide the probably highest amount of reliable information. For better clarity, only those two “extreme” types of media are compared regarding psychological outcomes. Due to the majority of the participants reporting more than just one type of media to obtain information about COVID-19, we could not compare different media types directly—instead, we compared the part of the sample using a specific type with the remaining part not using this type of media.

## Results

### Sample characteristics

70.4% of the participants were female (*N* = 4387), 28.8% male (*N* = 1793) and 0.9% reported to identify as diverse (*N* = 53). Mean age was 36.45 years (SD = 11.61, range 18–99). 17.2% of the sample had a secondary school degree or lower (*N* = 1072), 32.3% had a higher education entrance qualification (*N* = 2014) and 50.5% a university degree (*N* = 3147). 1052 participants reported to work in a medical context (16.9%). 664 participants suffered from a severe physical illness (10.7%). 18.0% of the sample reported having pronounced fears of physical diseases prior to the pandemic (*N* = 1124).

1667 participants knew someone diagnosed with COVID-19 (26.7%) and 1729 already suspected themselves to be infected (27.7%). 7.1% were currently under quarantine and 4.4% had been tested for COVID-19. Only 0.8% were diagnosed with COVID-19 (*N* = 50).

### Types of media

The majority of the participants reported to use primarily three different types of media formats to inform themselves about COVID-19 (32.1%; *N* = 1995). 25.1% reported two different types (*N* = 1563) and 20.6% used four different formats (*N* = 1286). 577 Participants stated to consume five types of media (9.3%) and 247 participants used six different types (4.0%;). In contrast, 9.1% of the sample reported to primarily use only one type of media to obtain information regarding the pandemic (*N* = 565).

**Fig. 2:**
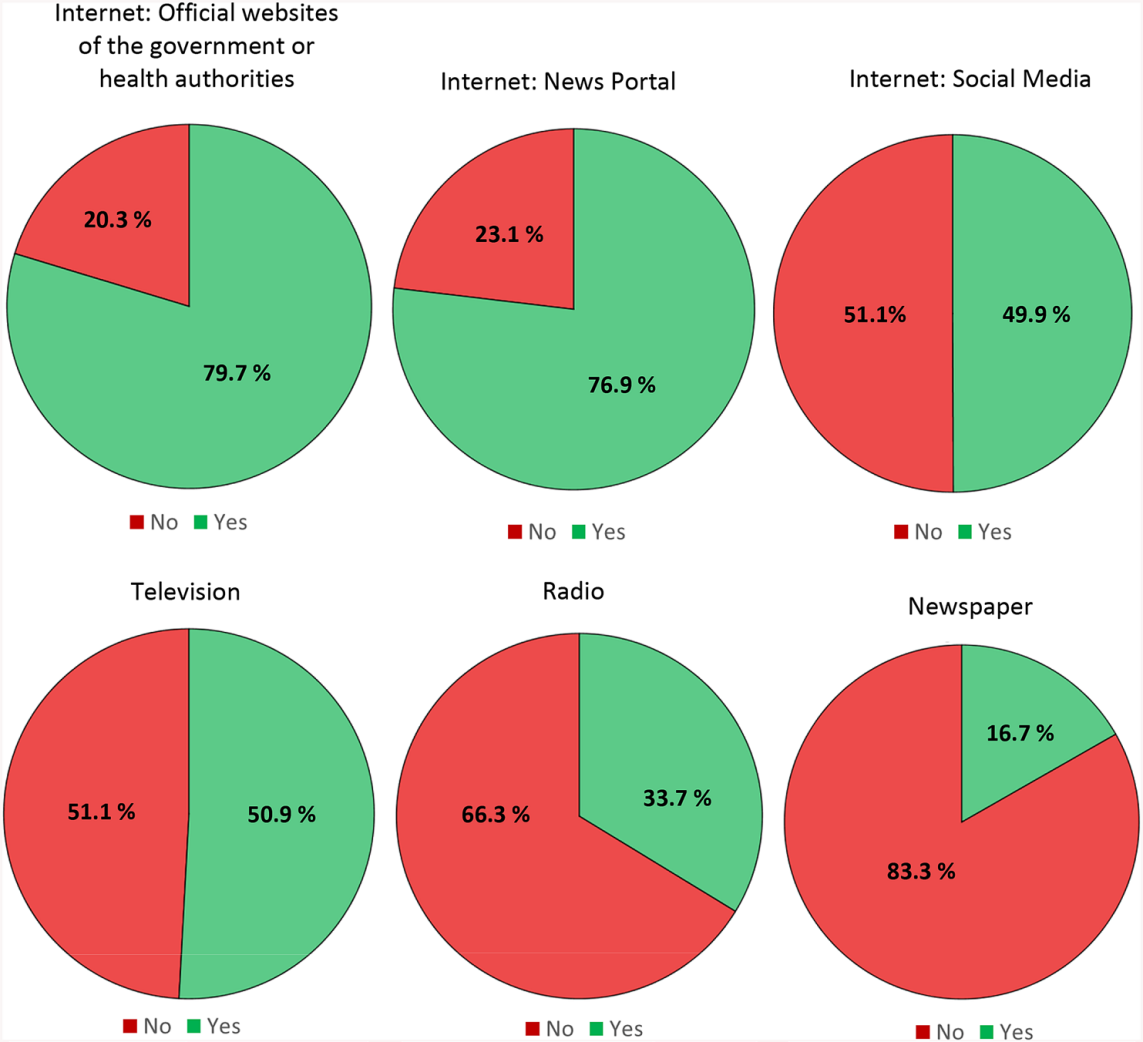
Primarily used types of media to obtain information about the COVID-19 pandemic (multiple answers were possible). Data based on *N* = 6233 participants

The most frequently used media format were official websites of the government or health authorities: 79.7% of the sample reported to use primarily such websites to get information about COVID-19 (*N* = 4967; see Fig. 2). The second most common format were online news portals (76.9%; *N* = 4792), followed by television, social media, radio, and newspapers. The percentage distribution is illustrated by Fig. 2.

### Duration and frequency

The reported daily average of media usage to get information regarding the COVID-19 pandemic was 2.40 h (SD = 2.01; range 0–24) and the Median was 2 h. The lowest 25% of the sample reported a usage of 1 h or less to obtain information about COVID-19 and the highest 25% reported 3 h or more. The mean frequency of media consumption was 7.23 times per day (SD = 28.76; range 0–999). The lowest 25% of the participants reported to check media 2 times per day or less and the highest 25% reported 6 or more times while the Median was 4.

### Duration and frequency of media usage and symptoms of anxiety and depression

The daily average time of media consumption was significantly positively correlated with anxiety symptoms regarding COVID-19, measured with the modified Specific-Phobia-Scale (*r* = 0.25 *p* < 0.001). The more time media was used to get information about COVID-19, the higher the anxiety symptoms were on average. The duration of media usage was as well significant positively correlated with the PHQ-4 total score (*r* = 0.21; *p* < 0.001) and both subscales (anxiety subscale GAD-2: *r* = 0.20; *p* < 0.001; depression subscale PHQ-2: *r* = 0.19; *p* < 0.001).

**Fig. 3:**
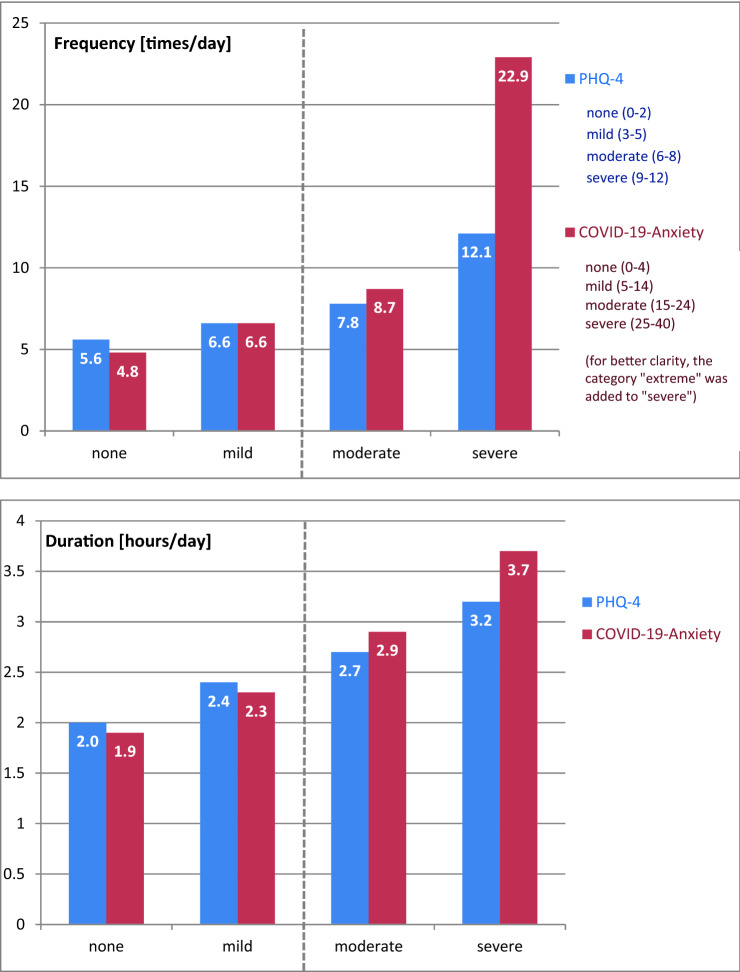
Severity of unspecific anxiety and depressive symptoms (PHQ-4) and COVID-19-related anxiety and the average amount of media consumption to ascertain information about COVID-19

The frequency of media usage was also significantly positively correlated with specific COVID-19 related fear (Phobia-Scale: *r* = 0.10; *p* < 0.001) and unspecific anxiety and depressive symptoms (PHQ-4: *r* = 0.09; *p* < 0.001; PHQ-2: *r* = 0.07; *p* < 0.001; GAD-2: *r* = 0.09; *p* < 0.001). Those associations of duration and frequency with the severity of specific and unspecific anxiety and depressive symptoms can also be seen in the descriptive examination of the means (see Fig. 3).

The more different types of media were used, the higher were the symptoms of specific fear, unspecific anxiety and depression (Phobia-Scale: *r* = 0.25; *p* < 0.001; PHQ-4: *r* = 0.32; *p* < 0.001; PHQ-2: *r* = 0.26; *p* < 0.001; GAD-2: *r* = 0.34; *p* < 0.001).

### Types of media and symptoms of anxiety and depression

Participants who reported the usage of official websites of the government or health authorities as a primary source of information showed significantly less unspecific anxiety and depression than participants who did not use primarily such websites (PHQ-4: *M* = 4.08, SD = 3.12 vs. *M* = 4.42, SD = 3.40; *F*(1, 6076) = 5.53, *p* = 0.019). In contrast, regarding specific COVID-19 related fear, participants who used official websites showed on average significantly more symptoms than participants not using official websites (*M* = 10.29, SD = 6.96 vs. *M* = 9.52, SD = 6.88; *F*(1, 6176) = 13.56, *p* < 0.001).

Participants who reported social media as a primary source of information showed—in comparison to participants not using social media—on average significantly more unspecific anxiety and depression (PHQ-4: *M* = 4.40, SD = 3.19 vs. *M* = 3.90, SD = 3.15; *F*(1, 6076) = 36.03, *p* = 0.019) and significantly more specific COVID-19 related anxiety symptoms (*M* = 10.64, SD = 6.97 vs. *M* = 9.62, SD = 6.89; *F*(1, 6176) = 33.11, *p* < 0.001).

### Pre-existing fears

Participants with pre-existing fears of physical diseases (prior to the pandemic) reported on average significantly more frequent (*M* = 10.56 vs. *M* = 6.49; *F*(1, 5173) = 18.70, *p* < 0.001) and longer (*M* = 2.82 vs. *M* = 2.30 h; *F*(1, 5173) = 205.39, *p* < 0.001) media consumption than participants without pre-existing fears. Moreover, the group with pre-existing fears scored higher in specific COVID-19 related fears (*M* = 14.14 vs. *M* = 9.25; *F*(1, 5173) = 470.59, *p* < 0.001) and unspecific anxiety and depressive symptoms (PHQ-4: *M* = 5.61 vs. *M* = 3.83; *F*(1, 5173) = 303.31, *p* < 0.001).

For participants who reported pre-existing fears, the correlations of frequency and duration of media exploitation with anxiety and depressive symptoms were even stronger than for the those without pre-existing fears: duration and PHQ-4: *r* = 0.25; *p* < 0.001 vs. *r* = 0.18; *p* < 0.001 (statistically significant difference of the coefficients, *z* = 1.89, *p* = 0.03); frequency and PHQ-4: *r* = 0.10; *p* = 0.001 vs. *r* = 0.08; *p* < 0.001 (not statistically significant difference, *z* = 0.79, *p* = 0.22).

Moreover, there was a significant interaction of pre-existing fears and usage of official websites (*F*(1, 6074) = 4.17, *p* = 0.041). In the group with pre-existing fears the PHQ-4 Score was slightly higher for people using official websites (*M* = 5.61 vs. *M* = 5.49), in the group without pre-existing fears the PHQ-4 was lower for people using official websites than for people not using official websites to obtain information about COVID-19. Regarding the interaction with social media as well as regarding specific COVID-19 related fear there were no significant effects depending on pre-existing fears.

### Reflection of media usage

The more time of media usage the participants reported, the higher was their subjective need to reduce their consumption of media because of its negative psychological consequences (*r* = 0.17; *p* < 0.001). Reductions that have been already made were significantly negatively correlated with the duration of current media consumption (*r* = − 0.08; *p* < 0.001). The frequency of media usage was also significantly but less strongly correlated with the need to reduce media consumption (*r* = 0.07; *p* < 0.001) and previously made reductions (*r* = − 0.03; *p* = 0.019).

## Discussion

First, our results of a positive correlation between COVID-19 related media exposure and the severity of unspecific anxiety, depression and topic-specific fear substantially agree with up-to-date findings of a web-based study from China. Using a comparable web-based cross-sectional design and language-validated versions of the seven-item generalized anxiety disorder scale (GAD-7) and the WHO-Five Well-Being Index (WHO-5), Gao and colleagues also found the severity of anxiety and depression to be significantly positively correlated with the frequency (determined ass “less”, “sometimes” or “frequently”) of self-reported media usage regarding COVID-19 within more than 4000 citizens [18]. In addition, the present study further demonstrates that not only the frequency but also—and even stronger—the daily duration as well as the diversity of media exposure are significantly positively correlated with global symptom severity and therefore emphasize the meaning of mental distress associated with COVID-19 media coverage, particularly once a “critical load” has been reached. This conclusion is supported by both a recent study that turned out media coverage as one of the strongest emotional stressors in the context of the current pandemic [17] and the data of the present trial. Although it is not clear if psychological strain is rather a consequence or a cause of more immoderate media exploitation, we found the frequency and the duration of COVID-19 related media exposure to be positively correlated with the subjective need for its reduction due to a substantial amount of topic-induced stress. This result may be interpreted in a way that a significant number of individuals are aware of “overconsumption” of information as a risk factor for mental wellbeing in the present crisis.

Therefore, a precise determination of risky media exploitation should be warranted to refine already existing recommendations in this field. We therefore ascertained concrete numeric values with respect to the frequency and the duration of media usage that are associated with at least mild to moderate symptom severity on the PHQ-4 and the DSM-5 Specific-Phobia-Scale. Our results indicate that the frequency with a possible hazardous association with mental health might be somewhat higher than the maximum of twice a day recommended by the WHO [10]. However, it seems to be important to note that we are able to identify COVID-19 related media usage of seven times or 2.5 h a day to be associated not only with critical values for unspecific anxiety and depression but also with COVID-19 specific fear. These results may provide some evidence for a distinct threshold of media consumption that is associated with an increased risk for a range of mental health complaints within a significant number of people.

Moreover, our results indicate that the amount of mental distress depends on the type of media primarily used for gaining information whereby the usage of social media was associated with a significantly higher degree of unspecific anxiety and depression than keeping informed by official sources. This finding supports the considerations of the WHO to primarily rely on information of health authorities for information about COVID-19 [10] and provide some evidence for a problematic role of social media in the context of mental health, probably due to an elevated stress level induced by a flood of unfiltered information and a more emotional style of communication. Interestingly, with regard to COVID-19 specific fear the usage of social media and official websites were both associated with significantly higher levels of fear compared to participants not using those media types. Although it cannot be ruled out that the favored sources of information may not be a cause but rather a result of psychological strain and/or other differences between the user groups, this finding may provide some evidence for a problematic role of both sources in case of prominent COVID-19 related fears.

Finally, we further assessed the impact of pre-existing health-related fears on the risk of psychological strain due to COVID-19 related media exposure and found both the frequency and the duration of media usage to be significantly stronger correlated to anxiety and depression in individuals with this characteristic than in those without. Although research regarding specific vulnerability for the development of psychopathology due to crisis-related media exposure is rare, some trials already focused on this topic in the context of posttraumatic stress. Results showed that the quality of emotional reaction during media exposure [15] or prior direct exposure to traumatic events [13, 14] were significantly associated with the risk for (acute) posttraumatic stress after topic-specific media consumption related to bombings or the attacks of September 11th, 2001. Our findings therefore provide further evidence that vulnerability, particularly those with relevance for the specific content of a disaster, lead to an increased probability of experiencing significant mental symptoms after crisis-related media exposure. Interestingly, participants with pre-existing health-related fears demonstrated a significantly higher frequency and duration of media exploitation as well as a substantially higher severity of (un)specific anxiety and depression than those without. This might be interpreted in a way that, probably related to dysfunctional checking behavior, vulnerable individuals slightly exceed the critical threshold of 2.5 h and 7 times daily that in turn leads to significant increased stress-related symptoms in these individuals. However, another explanation might be that this subgroup suffered from more severe anxiety and depression per se and the higher frequency/duration of media exposure is due to a more frequent safety behavior. Although causal relations cannot be proven due to the cross-sectional nature of this study, these findings may indicate that patients with health-related fears should pay special attention to their COVID-19 related media exposure to avoid extraordinary mental strain. Besides a pre-existing vulnerability for worrying about physical illness, a vulnerability for addictive behavior, respectively addictive internet/media use could be a potentially relevant factor regarding media consumption and COVID-19 related psychological parameters. Therefore it should be taken into account in future studies with validated and standardized questionnaires.

Several limitations of the present study have to be considered that may limit the generalization of our findings. Data sampling via social media might result in a major obstacle for some individuals to participate (e.g., for those who have limited access to or are not familiar with the use of social media such as the elderly). Addressing a convenience sample further might lead to a bias in a way that individuals experience a relatively high level of COVID-19 related psychosocial strain are more likely to take part in our study. Indeed, some evidence for sampling bias results from the fact that in relation to the general population an above-average portion of the participants was female and reported to work in the medical field. For reasons of feasibility, we used rather short questionnaires to assess for (un)specific anxiety and depression. The application of an unvalidated questionnaire to run for COVID-19 specific fear, however, represents a major limitation of this study. Finally, due to the cross-sectional nature of this trial, no causal conclusions can be drawn on this basis. However, due to the emerging situation, it should be important to gain an insight into the potential associations of media consumption and pandemic-related mental health as well as to compare available considerations of official authorities in this area with original data. As a consequence of our findings—especially if they will be confirmed in longitudinal studies—practical interventions for dealing with the current COVID-19 pandemic as well as with other possible crises in the future should be derived. For example, online intervention tools for people with critical media exploitation or a pronounced vulnerability for maladaptive psychological outcomes should be envisaged. Moreover, different media formats could include screening instruments for detecting a potentially problematical consumption of media and provide recommendations for a more adaptive usage of media reporting.

Summing up, our findings provide some evidence for a problematical role of media exposure in the context of the COVID-19 pandemic. The frequency/duration of media exploitation, type of media, the diversity of media usage as well as individual vulnerability might play an important role in the development of mental distress or might be a result of mental distress and therefore relevant in the perpetuation of mental distress. Since our data point to a distinct threshold of topic-specific media exposure that should be considered to avoid psychological strain, our results therefore further may serve as an orientation not only for the general population but also for professionals in the (mental) health sector to provide recommendations in this context. Future studies should try to clarify the causal relationship of our findings to create a reliable basis for considerations in this field.

## Data Availability

The questionnaire and the data of this study are available from the corresponding author upon reasonable request.
