# Correction to: Associations between COVID-19 related media consumption and symptoms of anxiety, depression and COVID-19 related fear in the general population in Germany

**DOI:** 10.1007/s00406-021-01290-8

**Published:** 2021-07-17

**Authors:** Antonia Bendau, Moritz Bruno Petzold, Lena Pyrkosch, Lea Mascarell Maricic, Felix Betzler, Janina Rogoll, Julia Große, Andreas Ströhle, Jens Plag

**Affiliations:** Department of Psychiatry and Psychotherapy, Campus Charité Mitte, Charité – Universitätsmedizin Berlin, corporate member of Freie Universität Berlin and Humboldt Universität zu Berlin, Charitéplatz 1, 10117 Berlin, Germany

## Correction to: European Archives of Psychiatry and Clinical Neuroscience (2021) 271:283–291 10.1007/s00406-020-01171-6

The article “Associations between COVID-19 related media consumption and symptoms of anxiety, depression and COVID-19 related fear in the general population in Germany”, written by Antonia Bendau, Moritz Bruno Petzold, Lena Pyrkosch, Lea Mascarell Maricic, Felix Betzler, Janina Rogoll, Julia Große, Andreas Ströhle and Jens Plag was originally published electronically on the publisher’s internet portal on July 20, 2020 without open access. With the author(s)’ decision to opt for Open Choice the copyright of the article changed to © The Author(s) 2020 and the article is forthwith distributed under a Creative Commons Attribution 4.0 International License, which permits use, sharing, adaptation, distribution and reproduction in any medium or format, as long as you give appropriate credit to the original author(s) and the source, provide a link to the Creative Commons licence, and indicate if changes were made. The images or other third party material in this article are included in the article’s Creative Commons licence, unless indicated otherwise in a credit line to the material. If material is not included in the article’s Creative Commons licence and your intended use is not permitted by statutory regulation or exceeds the permitted use, you will need to obtain permission directly from the copyright holder. To view a copy of this licence, visit http://creativecommons.org/licenses/by/4.0/. Open Access funding enabled and organized by Projekt DEAL.

The original article has been updated.

